# Blockade of ARHGAP11A reverses malignant progress via inactivating Rac1B in hepatocellular carcinoma

**DOI:** 10.1186/s12964-018-0312-4

**Published:** 2018-12-13

**Authors:** Bin Dai, Xuan Zhang, Runze Shang, Jianlin Wang, Xisheng Yang, Hong Zhang, Qi Liu, Desheng Wang, Lin Wang, Kefeng Dou

**Affiliations:** Department of Hepatobiliary Surgery, Xijing Hospital, The Fourth Military Medical University, Xi’an, China

**Keywords:** ARHGAP11A, Hepatocellular carcinoma, EMT, Metastasis, Rac1B

## Abstract

**Background:**

The molecular signaling events involving in high malignancy and poor prognosis of hepatocellular carcinoma (HCC) are extremely complicated. Blockade of currently known targets has not yet led to successful clinical outcome. More understanding about the regulatory mechanisms in HCC is necessary for developing new effective therapeutic strategies for HCC patients.

**Methods:**

The expression of Rho GTPase-activating protein 11A (ARHGAP11A) was examined in human normal liver and HCC tissues. The correlations between ARHGAP11A expression and clinicopathological stage or prognosis in HCC patients were analyzed. ARHGAP11A was downregulated to determine its role in the proliferation, invasion, migration, epithelial-to-mesenchymal transition (EMT) development, and regulatory signaling of HCC cells in vitro and in vivo.

**Results:**

ARHGAP11A exhibited high expression in HCC, and was significantly correlated with clinicopathological stage and prognosis in HCC patients. Moreover, ARHGAP11A facilitated Hep3B and MHCC97-H cell proliferation, invasion, migration and EMT development in vitro. ARHGAP11A knockdown significantly inhibited the in vivo growth and metastasis of HCC cells. Furthermore, ARHGAP11A directly interacted with Rac1B independent of Rho GTPase- activating activity. Rac1B blockade effectively interrupted ARHGAP11A-elicited HCC malignant phenotype. Meanwhile, upregulation of Rac1B reversed ARHGAP11A knockdown mediated mesenchymal-to-epithelial transition (MET) development in HCC cells.

**Conclusion:**

ARHGAP11A facilitates malignant progression in HCC patients via ARHGAP11A-Rac1B interaction. The ARHGAP11A/Rac1B signaling could be a potential therapeutic target in the clinical treatment of HCC.

**Electronic supplementary material:**

The online version of this article (10.1186/s12964-018-0312-4) contains supplementary material, which is available to authorized users.

## Background

Hepatocellular carcinoma (HCC), accounting for 90% of primary liver cancer cases, is one of the most prevalent cancers worldwide and exhibits high mortality [1, 2]. The poor prognosis of HCC is mainly associated with the high frequency of late-stage disease, recurrence and metastasis. In addition, HCC metastasis is specially characterized by de novo nodule formation in liver parenchyma and portal vein invasion, which contributes to 90% of all tumor-related deaths [3, 4]. Therapeutic strategies aiming at HCC survival improvement have not yet achieved a satisfactory outcome. This is due, in part, to our inability to identify in advance the molecular mechanism of HCC in patients who are at high risk of cancer metastasis. Hence, more elucidation about HCC signal pathways in malignancy regulation may help to identify novel effective molecular targets for HCC treatment.

Rho GTPase-activating proteins (RhoGAPs) have been thought to activate Rho GTPases originally, and to act as tumor suppressors [5]. RhoGAPs generally link cell migration and proliferation pathways, and are frequently downregulated in cancers [6]. Furthermore, studies have demonstrated that RhoGAPs could negatively regulate epithelial-to-mesenchymal transition (EMT) as critical modulators, thus influencing the progress and outcome of EMT relevant diseases. For instance, Rich1 was found to negatively regulate the epithelial cell cycle, proliferation and adhesion via the CDC42/RAC1-PAK1-Erk1/2 pathway [7]. ARHGAP29 could inactivate the RhoA-ROCK1 EMT pathway to reduce fibrosis and ameliorate intrauterine adhesions [8]. A STARD13-correlated ceRNA network inhibited EMT and metastasis of breast cancer in vitro and in vivo [9].

ARHGAP11A, an uncharacterized RhoGAP, localizes to the plasma membrane in early mitosis and to the equatorial membrane in anaphase, and is known as a regulator of cell cycle-dependent motility. Unlike typical RhoGAPs members, Kagawa Y et al. [10] identified that ARHGAP11A dynamically regulated colon cancer cell motility and invasion in vivo. Researchers have also found that ARHGAP11A could directly interact with p53 tetramerization domain to exhibit a Rho-independent role in cancer [6]. Recently, the expression of ARHGAP11A was found in human BLBC cell lines, and ARHGAP11A knockdown caused CDKN1/p27-mediated arrest in G1 phase of the cell cycle [11]. Hence, the molecular mechanism involving in RhoGAPs regulation network is relatively complicated, and further investigation is needed to reveal their precise roles in different diseases.

In the present study, we focused on the expression and specific function of ARHGAP11A in HCC and unraveled a new therapeutic paradigm of HCC inhibition via ARHGAP11A blockade. Furthermore, the ARHGAP11A-Rac1B interaction brought us a novel potential therapeutic target for HCC patients.

## Methods

### Patients and tissue specimens

Seventy-five HCC patients who underwent curative resection at the Department of Hepatobiliary Surgery, Xijing Hospital, Fourth Military Medical University (Xi’an, China) were enrolled in the study. None of these patients had received chemotherapy, ethanol injection, radiofrequency ablation, or transarterial chemoembolization before surgical resection. All patients met the diagnostic criteria of the American Association for the Study of Liver Diseases. The study protocol was approved by the Ethics Committee of Xijing Hospital. Written informed-consent was obtained from each patient or from his/her legal guardians.

### Cell lines and culture conditions

The HCC cell lines Hep3B and Hep1–6 (obtained from the Cell Bank of Type Culture Collection of the Chinese Academy of Sciences), and MHCC97-H (obtained from the Liver Cancer Institute of Fudan University) were cultured at 37 °C in a humidified 5% CO_2_ atmosphere and DMEM supplemented with 10% fetal bovine serum (HyClone, Logan, UT, USA).

### Plasmids and cell transfection

Lentiviral plasmids encoding ARHGAP11A or a negative control were designed and produced by Genechem (Shanghai, China). Hep3B and MHCC97-H cells were grown in 6-well plates to 20–30% confluence, and the culture medium was replaced with transduction enhancing solution containing lentivirus at a MOI of 20. After 24 h, the medium was replaced with complete medium, and the cells were cultured for 72 h. Then, the cells were selected with 1 μg/ml puromycin for 3 days and harvested for subsequent studies. The Rac1B-siRNA and Scrambled were designed by Genechem (Shanghai, China), and the transfection process was performed according to the manufacturer’s instructions. The Rac1B plasmid and empty vector were purchased from Genecreate (Wuhan, China). Relevant sequences, ARHGAP11A-Sh: GTATCAGTTCACATCGATA, ARHGAP11A human scrambled: TTCTCCGAACGTGTCACGT, ARHGAP11A-Lentivirus-1: CAGCAGCAATCTTGCAGTAAT, ARHGAP11A-Lentivirus-2: GAGCAGTCATCAGTAACAAAT, ARHGAP11A mouse scrambled: AAGCACAGAAGTCTACGTCTT, Rac1B-Si: UGGAGACACAUGUGGUAAAGAUAGA, Rac1B human scrambled: CGUACGCGGAAUACUUCGATTAGA.

### Construction of tissue microarrays and immunohistochemistry

A total of 75 paraffin-embedded HCC samples and their corresponding adjacent liver tissues were used to construct a tissue microarray (Shanghai Outdo Biochip Co., LTD. Shanghai, China). The tissue microarray was used to detect ARHGAP11A expression by immunohistochemistry. Briefly, the slide was routinely deparaffinized, hydrated, boiled in 10 mM citrate buffer (pH 6.0) for antigen retrieval and maintained at a sub-boiling temperature for 10 min. Endogenous peroxidases were inactivated by incubating the slide in 3% hydrogen peroxide for 10 min. Next, we used 5% normal goat serum to block the slide for 1 h at room temperature. The tissue sections were incubated with a primary antibody at 4 °C overnight. After washing with PBS, the slide was incubated with a biotinylated secondary antibody at room temperature for 1 h and treated with diaminobenzidine (DAB kit, ZSGB-BIO, China) for approximately 10 min. Hematoxylin was used to counterstain the sections.

### Quantitative RT-PCR analysis

Total RNA was extracted from tissues or cultured cells with RNAiso Plus (TaKaRa, Dalian, China) for mRNA analysis. A total of 400 ng of RNA was subjected to reverse transcription to generate cDNA. To quantify mRNA expression, qRT-PCR was performed using a SYBR Premix Ex Taq™ II kit (TaKaRa, Dalian, China) in accordance with the manufacturer’s instructions. β-actin was used as the reference gene. The relative fold changes in the mRNA levels were calculated using the 2-^ΔΔ^CT method. All samples were analyzed in triplicate. The primer sequences are shown in Additional file 1: Table S1.

### Western blot analysis

Whole cell extracts were harvested in RIPA lysis buffer (Beyotime, Shanghai, China) containing protease inhibitors (Thermo Scientific, Rockford, IL, USA) and phosphatase inhibitors (Thermo Scientific). Western blot analysis was performed using standard procedures as previously described [2]. The antibodies used for western blotting are listed in Additional file 2: Table S2. The protein bands were detected with a ChemiDoc™XRS+ and Image Lab TM software (Bio-Rad, Hercules, CA, USA).

### Cell proliferation and colony formation assays

Cell proliferation was analyzed with a Cell Counting Kit-8 (CCK-8) assay. Five replicate cell samples were seeded into 96-well plates at a density of 1.5 × 10^3^ (Hep3B) and 2.0 × 10^3^ (MHCC97-H) cells/well. At the indicated time points (0, 1, 2, 3, 4 and 5 d), 90 μl of culture medium containing 10% serum and 10 μl of CCK-8 solution mix was added to each well. Following incubation for 2 h at 37 °C, the absorbance was measured at 450 nm using a spectrophotometer.

For colony formation assay, cells were seeded at a density of 300 cells/well in a 6-well plate and cultured in 2 ml of DMEM supplemented with 10% FBS for 2 weeks. Then, the colonies containing over 50 cells were fixed in 95% ethanol and stained with a 4 g/L crystal violet solution for counting.

### Cell cycle analysis

Hep3B and MHCC97-H cells transfected with shRNA targeting human-specific genes were grown in 6-well plates. After 48 h, the cells were resuspended in 200 μl of PBS, and 1 ml of a 70% ethanol solution was added dropwise while stirring. Then, the cells were stained with 50 μg/ml propidium iodide in PBS plus 100 μg/ml RNase for 15 min at 37 °C and analyzed for DNA content using a CyAn ADP flow cytometer. Cell cycle analysis was performed for 10,000 events using the ModFit LT™ software.

### Cell apoptosis analysis

Cell apoptosis was detected using an Annexin V-PI detection kit and flow cytometry (FCM). Hep3B and MHCC97-H cells transfected with shRNA targeting human-specific genes were grown in 6-well plates. After 48 h, the cells were harvested for Annexin V-PI staining according to the manufacturer’s instructions (BD Biosciences Pharmingen). The cells were analyzed by flow cytometry, and early and late apoptotic cells were measured.

### Cell invasion and migration assays

Cell migration was evaluated with a wound healing assay. For the wound healing assay, 5.0× 10^5^ cells transfected with shRNA or NC were grown to confluence on 35-mm plates, and a wound was made in the monolayer with a sterile pipette tip. The cells were washed twice with PBS to remove debris, and fresh medium was added. Phase-contrast images of the wounded area were taken at 0 and 24 h after wounding. The results were shown as percent of wound healing (=1- blank area at 24 h/ blank area at 0 h), and analyzed by NIH image J Software. The cell invasion assay was performed with Transwell Permeable Supports (8 μm pore size; Millipore) with Matrigel (BD Biosciences, San Jose, CA, USA) matrix. Cells (0.5 × 10^5^/chamber) in culture medium (200 μl) without serum were introduced to the upper side of the chamber. Culture medium (600 μl) containing 10% serum was used as a chemoattractant. The plates were incubated for 24 h at 37 °C in 5% CO_2_. Then, the cells that had invaded the lower surface of the membrane were fixed with 95% ethanol and stained with a 4 g/L crystal violet solution. Cells on the upper surface of the membrane were removed with cotton swabs. Cells adhering to the underside of the membrane were counted in five randomly selected areas under a 100× microscope field. All experiments were performed at least three times.

### Tumor growth and metastasis experiments in vivo

To observe the effects of ARHGAP11A on tumor growth in vivo, 2 × 10^6^ MHCC97-H cells stably expressing shRNA against ARHGAP11A were suspended in 150 μl of PBS and subcutaneously injected into nude mice. The mice were examined for tumor formation for 30 days and were euthanized. Tumors were harvested and weighed at the end of the experiment. To investigate experimental lung metastasis, luc-ARHGAP11A-lentivirus-2-transfected Hep1–6 cells (2.5 × 10^6^) were suspended in 150 μl of PBS and injected into C57BL mice through the tail vein. After 5 weeks, the mice were anesthetized and intraperitoneally injected with 150 μg/body weight (g) D-luciferin (Caliper, Hopkinton, MA, USA). Fifteen minutes later, the bioluminescence from each mouse was imaged in an IVIS Lumina II Imaging System (Caliper). All experimental procedures involving mice were performed in accordance with the Guide for the Care and Use of Laboratory Animals and were approved by The Research Animal Care and Use Committee of Fourth Military Medical University.

### Co-immunoprecipitation (co-IP)

Co-immunoprecipitation was carried out as described previously [12]. Briefly, cells were lysed with RIPA buffer with DNase and protease inhibitors for 30 min at 21 °C. The total protein concentrations were measured with BCA assay. Rabbit anti-ARHGAP11A polyclonal antibody was incubated with protein A beads at 4 °C for 1 h and conjugated to the beads with 450 μM DSS solution following the manufacturer’s protocol. Then, ARHGAP11A and its interacting proteins were purified with the antibody conjugated beads, follow by Western blotting. For Co-IP of endogenously expressed ARHGAP11A and Rac1B, cultured human cells transfected with HA-tagged Rac1B were lysed and then followed the procedure as mentioned above.

### Co-immunoprecipitation coupled with mass spectrometry (CoIP-MS)

Co-immunoprecipitation was firstly performed as described above. Then, the purified ARHGAP11A and its interacting proteins were processed following the manufacturer’s protocol and analyzed through mass spectrometric using LC-MS/MS (Ekspert TM nanoLC; AB Sciex TripleTOF™ 5600^+^).

### RhoA, Rac1 and Rac1B activity assays

Total and GTP-bound RhoA, Rac1 and Rac1B levels were measured as previously described [13]. Briefly, HCC cells treated with or without ARHGAP11A-Sh were lysed in 400 μl of a lysis buffer containing 20 μg of GST-CRIB (for Rac1 and Rac1B) or GST-Rhotekin (for RhoA). The lysates were centrifuged at 20,000 g for 10 min, and the supernatants were incubated with glutathione-Sepharose (20 μl) for 1 h at 4 °C. Glutathione-Sepharose was precipitated by centrifugation, and the bound proteins were probed with anti-RhoA, anti-Rac1, anti-Rac1B antibodies.

### Statistical analysis

All of the statistical analyses were performed using SPSS version 22.0 (SPSS, Chicago, IL, USA) and GraphPad Prism 6.0(GraphPad software, La Jolla, CA, USA). Data were presented as the mean ± SD of at least three independent experiments or multiple independent mice as indicated. Student’s *t*-test (two-tailed) was used with a *P* value < 0.05. Kaplan-Meier survival data were reanalyzed using the log-rank test. The Mann–Whitney U-test and Spearman’s rank correlation test were used when appropriate.

## Results

### Expression and clinical signification of ARHGAP11A in HCC patients

**Fig. 1:**
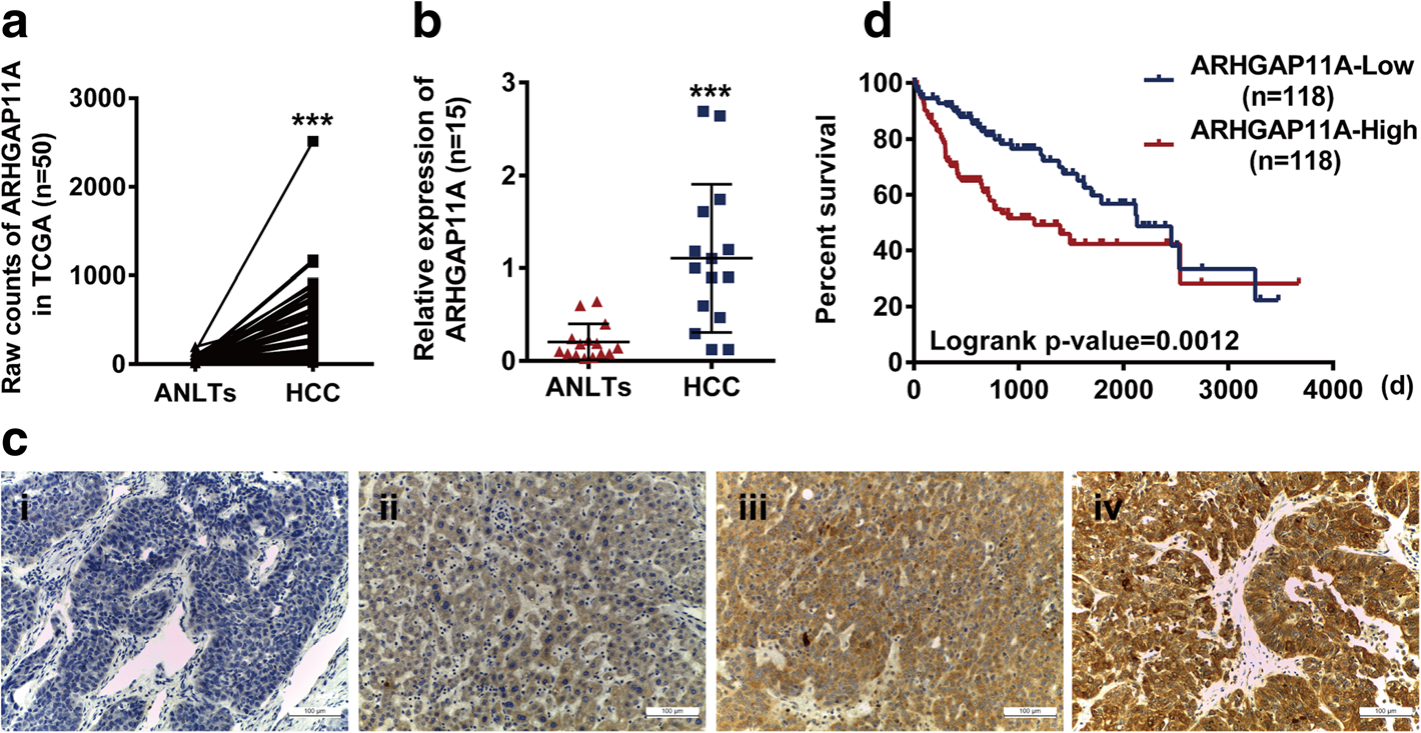
Overexpression of ARHGAP11A is associated with worse clinical outcome in HCC. **a** RNA-Seq data from TCGA (*n* = 50) was analyzed to explore ARHGAP11A expression in HCC tissues and ANLTs (adjacent non-tumor liver tissues). ***, *P* < 0.001 versus ANLTs. **b** The mRNA expression of ARHGAP11A in HCC tissues or ANLTs was further verified (*n* = 15). ***, *P* < 0.001 versus ANLTs. **c** The protein expression of ARHGAP11A in the human tissue array was detected by immunohistochemistry staining (*n* = 15). Representative photos are shown. i, negative, ii, weak, iii, moderate and iv, strong. Scale bars, 100 μm. **d** Comparison of the survival of patients with high ARHGAP11A expression (ARHGAP11A-High, *n* = 118) and low ARHGAP11A expression (ARHGAP11A-Low, *n* = 118) using the Kaplan-Meier method

To examine the expression of ARHGAP11A in HCC, we analyzed RNA-Seq data from The Cancer Genome Atlas (TCGA) Project. Strikingly, ARHGAP11A was found to be expressed in most HCC tissues compared with normal-like tissues and exhibited different levels (Fig. 1a). This conclusion was consistent with our qRT-PCR mRNA results (Fig. 1b). We further detected ARHGAP11A protein expression in tissues from HCC patients with immunohistochemistry. As shown in Fig. 1c, different levels of ARHGAP11A expression were observed in different HCC tissues. The correlation statistics between ARHGAP11A expression and clinicopathological characteristics in HCC patients are shown in Table 1. High expression of ARHGAP11A was identified to be correlated with tumor size, differentiation, metastasis and TNM stage but not with other clinicopathological characteristics, such as gender, age, and AFP in patients with HCC.

**Table 1:** The correlation between ARHGAP11A expression and clinicopathological characteristics in HCC patients

Clinicopathological variables	ARHGAP11A expression		*P* value	χ^2^
	High	Low		
Gender			*P* > 0.05	0.3
Male	33	29		
Female	8	5		
Age			*P* > 0.05	0.574
< 45 y	18	12		
≥ 45 y	23	22		
Maximal tumor size			*P* < 0.05	4.467
≤ 5 cm	19	24		
> 5 cm	22	10		
Tumor differentiation			*P* < 0.05	10.941
I-II	20	29		
III-IV	21	5		
Serum AFP			*P* > 0.05	0.313
≤ 400 (ng/ml)	12	8		
> 400 (ng/ml)	29	26		
Metastasis			*P* < 0.05	3.969
No	30	31		
Yes	11	3		
TNM stage			*P* < 0.001	17.844
I-II	9	24		
III	32	10		

Statistically significant (*P* < 0.05)

Moreover, the TCGA dataset was utilized to explore the clinical significance of ARHGAP11A mRNA expression. The results indicated that the mRNA level of ARHGAP11A was correlated with the clinical grade, TNM stage and pathological stage of HCC (Table 2). In addition, Kaplan-Meier survival analysis revealed that HCC patients with low ARHGAP11A expression exhibited prolonged survival. The median overall survival was 2131 and 1149 days in patients with low and high ARHGAP11A expression, respectively (Fig. 1d). Taken together, these results show that ARHGAP11A, part of the RhoGAP family, whose members had been presumed to act as suppressors and to be deleted in cancer, is upregulated and might serve as an oncogene in HCC.

**Table 2:** The mRNA level of ARHGAP11A correlated to clinicopathological variables in TCGA

Clinicopathological variables	ARHGAP11A mRNA expression		Total	*P* value
	Low	High		
Clinical grades				
G1/2	137	95	232	.000
G3/4	47	87	134	
Total	184	182	366	
T Stage				
T1	106	75	181	.001
T2	41	53	94	
T3/4	36	57	93	
Total	183	185	368	
Pathological Stage				
Stage I	99	72	171	.004
Stage II	39	47	86	
Stage III/IV	36	54	90	
Total	174	173	347	

### ARHGAP11A is indispensable for HCC cell proliferation, but not apoptosis, in vitro

**Fig. 2:**
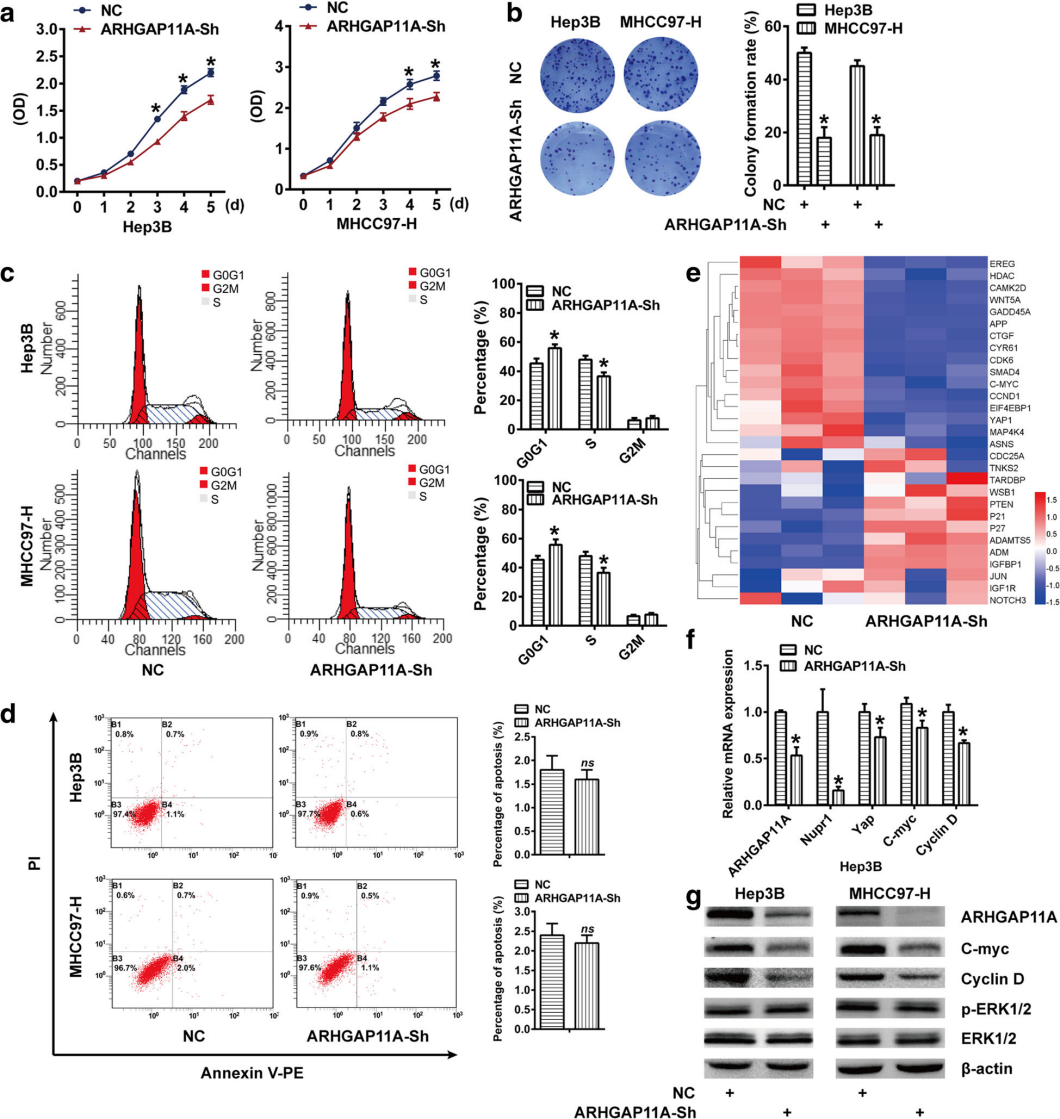
ARHGAP11A is indispensable for HCC cell proliferation, but not apoptosis, in vitro. Columns or curves, mean (*n* = 3, in triplicate); bars, SD. *, *P* < 0.05 versus NC. *ns*, no significance. **a** Hep3B and MHCC97-H cells were stably infected with Scrambled (NC) or ARHGAP11A-Sh and cultured for 1–5 days. Cell proliferation was detected by a CCK8 assay. **b** The colony forming ability of Hep3B and MHCC97-H cells following ARHGAP11A knockdown. **c** ARHGAP11A knockdown arrested the cell cycle in G0/G1 phase. **d** Flow cytometry was used to assess apoptosis in ARHGAP11A-Sh cells or NC. **e** Heatmap showing the differential expression of genes involved in cell proliferation upon ARHGAP11A knockdown (*n* = 3, triplicate). **f** Relative mRNA expression of ARHGAP11A, Nupr1, Yap, C-myc and Cyclin D determined by qRT-PCR analysis in ARHGAP11A-Sh or NC Hep3B cells. **g** Protein expression of ARHGAP11A, C-myc, Cyclin D, p-ERK1/2 and ERK1/2 in one experiment, representative of three

The RhoGAPs family is thought to inhibit cell cycle progression and induce apoptosis in various cancers [14, 15]. To assess the specific function of ARHGAP11A in HCC, ARHGAP11A was genetically ablated to determine its role in cell proliferation and apoptosis. ARHGAP11A expression was silenced in two human HCC cell lines, Hep3B (low malignancy) and MHCC97-H (high malignancy) with lentivirus-delivered shRNA constructs. The effectiveness of ARHGAP11A silencing was confirmed by qRT-PCR and western blot (Additional file 3: Figure S1). CCK-8 and colony formation assays showed that ARHGAP11A deletion significantly decreased the proliferation of both HCC cell lines (Fig. 2a, b). These results suggest that ARHGAP11A is indispensable for HCC cell proliferation and positively regulates cell growth. Moreover, to further investigate the decreased proliferation of ARHGAP11A-knockdown cells, we subsequently performed flow cytometry analysis to detect cell cycle progression and the apoptosis level. ARHGAP11A deficiency increased the percentage of cells in G0/G1 phase and decreased the percentage of cells in S phase (Fig. 2c). Curiously, no difference was found in the cell apoptosis level (Fig. 2d).

In addition, we compared the genome-wide expression pattern of Hep3B cells transduced with ARHGAP11A-shRNA with that of empty vector transfected Hep3B cells (NC). We identified 255 upregulated genes and 179 downregulated genes in Hep3B cells following ARHGAP11A knockdown (Data not shown). As shown in Fig. 2e, most genes involved in cell proliferation were downregulated by ARHGAP11A silencing. Considering that C-myc and CyclinD, among others, are G1/S transition regulators, we analyzed their mRNA and protein expression with qRT-PCR and western blot, respectively. Consistent with microarray results, Nupr1, Yap, C-myc and CyclinD were downregulated after ARHGAP11A knockdown (Fig. 2f, g and Additional file 4: Figure S2). These data strongly suggest that ARHGAP11A may regulate HCC cell proliferation via C-myc and Cyclin D. Though ARHGAP11A has been reported to interact with p53 to induce human glioma U87 cell apoptosis, previously [6], our results indicate that ARHGAP11A does not affect p53 expression (Additional file 5: Figure S3) or cell apoptosis in HCC.

### ARHGAP11A facilitates HCC cell invasion, migration and EMT

**Fig. 3:**
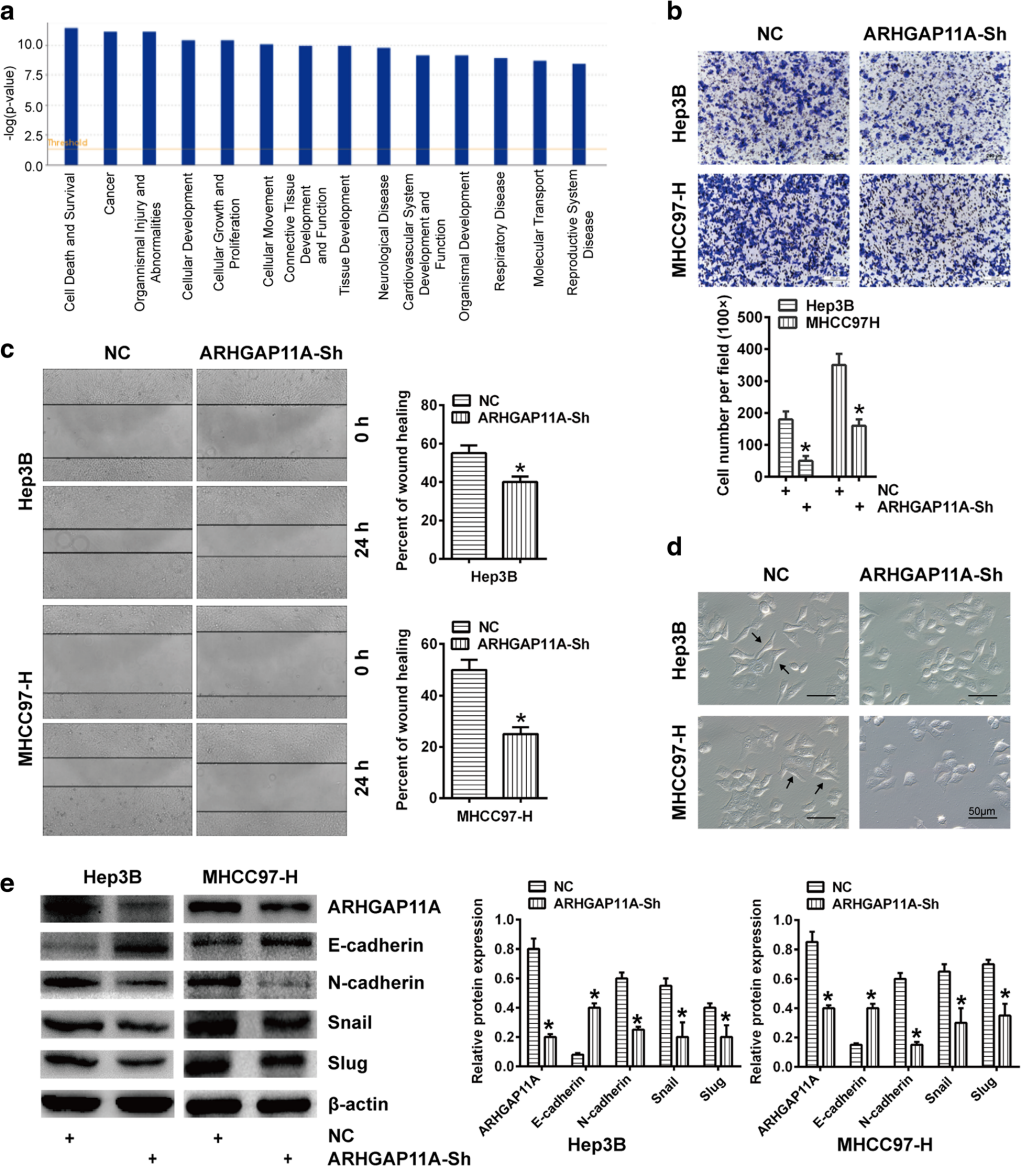
ARHGAP11A facilitates HCC cell invasion, migration and EMT. Columns, mean (*n* = 3, in triplicate); bars, SD. *, *P* < 0.05 versus NC. **a** Ingenuity Pathway Analysis (IPA) showed the functional pathways that were downregulated in Hep3B cells upon ARHGAP11A suppression. The orange line represents the cutoff value for significance. **b** Invasion ability of ARHGAP11A- knockdown HCC cells. Representative photos of the Transwell assay are shown. **c** Migration ability of ARHGAP11A-knockdown HCC cells. Representative photos of the wound healing assay are shown. **d** Representative microscopic images captured with inverted fluorescence microscope (OLYMPUS-DP72, Japan) are shown for the morphological changes of ARHGAP11A-knockdown HCC cells. Arrowheads, spindle-shaped cells. Scale bars, 50 μm. **e** E-cadherin, N-cadherin, Snail and Slug expression in ARHGAP11A-knockdown HCC cells was measured by western blot

To better understand the molecular mechanism of ARHGAP11A action in HCC cells, we compared the global gene expression of Hep3B shARHGAP11A cells with that of the corresponding control cells. Pathway analyses generated using Ingenuity Pathway Analysis (IPA) software identified the major functionally related gene groups that were differentially expressed in shARHGAP11A cells compared with control cells. Pathways implicated in cellular development, cell growth and proliferation, and cellular movement, among others, were mostly suppressed (Fig. 3a).

To characterize the action of ARHGAP11A on the HCC malignant phenotype, we investigated the effect of ARHGAP11A ablation on HCC cell invasion and migration. ARHGAP11A silencing inhibited the invasion capacity of HCC cells, as indicated by a Transwell assay (Fig. 3b). In addition, migration ability was also confirmed to be restricted following ARHGAP11A knockdown via a wound healing assay (Fig. 3c). Emerging evidence has shown that EMT is frequently activated and facilitates cell invasion and migration in cancers [16, 17]. To investigate whether EMT was activated to promote ARHGAP11A-induced invasion and migration in HCC, we observed morphological changes and the expression of EMT phenotype markers in ARHGAP11A-knockdown HCC cells. ARHGAP11A-knockdown recovered HCC cell’s polarity and cell-cell adhesion from a motile, multipolar, and spindle-shaped cell phenotype (Fig. 3d). Expression of the epithelial marker E-cadherin increased, while expression of the mesenchymal markers N-cadherin and Snail decreased in ARHGAP11A-knockdown cells (Fig. 3e). Taken together, these results show that ARHGAP11A activates EMT in HCC cells and facilitates cell invasion and migration in vitro.

### ARHGAP11A deletion suppresses in vivo growth and metastasis of tumor xenografts

**Fig. 4:**
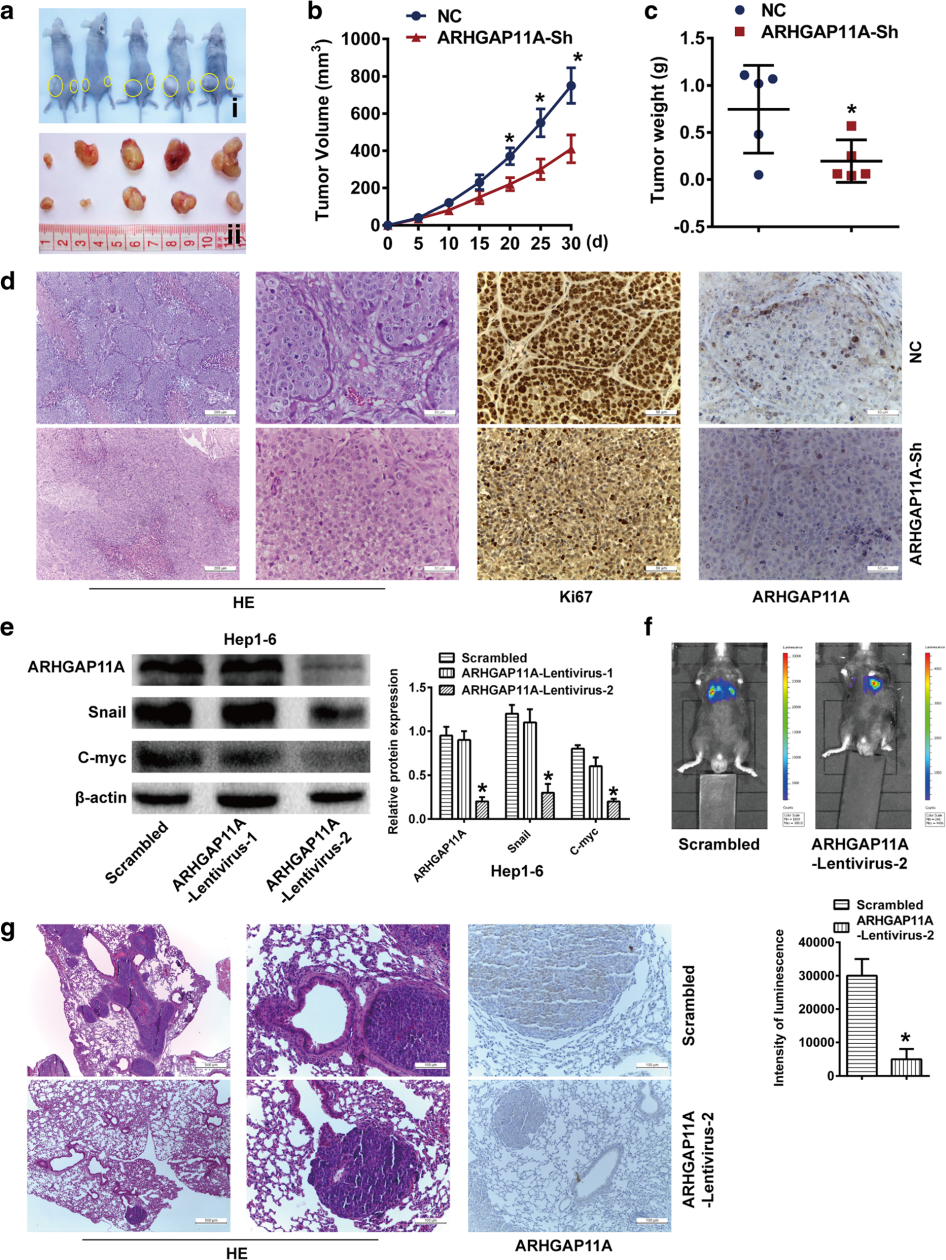
ARHGAP11A accelerates HCC tumor growth and metastasis in vivo. **a** ARHGAP11A-Sh-transfected MHCC97-H cells were injected subcutaneously into nude mice (*n* = 5). After 30 days, all mice were euthanized, and tumors were excised. Representative tumor photos are shown. i, NC left, and ARHGAP11A-Sh right. ii, NC up and ARHGAP11A-Sh down. **b** Growth curve of tumor volumes. *, *P* < 0.05 versus NC. **c** Tumor weight. *, *P* < 0.05 versus NC. **d** Representative images of tumor HE staining, and Ki-67 and ARHGAP11A immunohistochemistry staining are shown. Scale bars, 200 μm and 50 μm, respectively. **e** ARHGAP11A expression in Hep1–6 cells transduced with ARHGAP11A-lentivirus or a scrambled construct was analyzed by western blotting. **f** Representative bioluminescence images of C57BL mice 5 weeks after injection with cells transduced with ARHGAP11A-lentivirus or a scrambled construct are shown (*n* = 7). The luminescence intensity of lung metastases from luc-ARHGAP11A-Lentivirus-2- or scramble-transfected Hep1–6 cells was different. *, *P* < 0.05 versus the scrambled construct. **g** Lung metastatic nodules and ARHGAP11A expression in metastatic nodules were investigated with HE and immunohistochemical staining. Representative images are shown. Scale bars, 500 μm and 100 μm, respectively

To further evaluate the effect of ARHGAP11A knockdown on HCC cell growth in vivo, ARHGAP11A- knockdown MHCC-97H cells were injected into the flanks of nude mice to form xenograft tumors (Fig. 4a), and the tumor growth rate was monitored. Both the tumor volume and weight of mice in the ARHGAP11A- knockdown group were markedly lower than those of mice in the negative control group (Fig. 4b, c). Moreover, Ki-67 and ARHGAP11A staining were performed to detect the proliferation of tumor cells and the expression of ARHGAP11A. Tumors derived from ARHGAP11A- knockdown cells exhibited much weaker staining of Ki-67 and ARHGAP11A (Fig. 4d). Thus, our results support the hypothesis that ARHGAP11A has an oncogenic role, rather than a tumor suppressive role, in HCC cells.

To evaluate tumor metastasis, Hep1–6 cells transduced with ARHGAP11A-lentivirus were chosen. As shown in Fig. 4e, ARHGAP11A-lentivirus-2 showed a relatively higher efficacy in inhibiting ARHGAP11A expression, along with Snail and C-myc expression. Luciferase-labeled Hep1–6 cells were transduced with ARHGAP11A-lentivirus-2 or a scrambled construct and injected into C57BL mice intravenously. Five weeks later, the bioluminescence of lung metastases in the ARHGAP11A-lentivirus-2 group was weaker than that of the control group (Fig. 4f). Representative hematoxylin and eosin (HE) staining of metastatic lung nodules is shown in Fig. 4g. ARHGAP11A staining was also observed in lung metastatic nodules, consistent with our in vitro results. Taken together, these results show that ARHGAP11A knockdown impairs the growth and metastasis of HCC cells in vivo.

### ARHGAP11A interacts with Rac1B in HCC

**Fig. 5:**
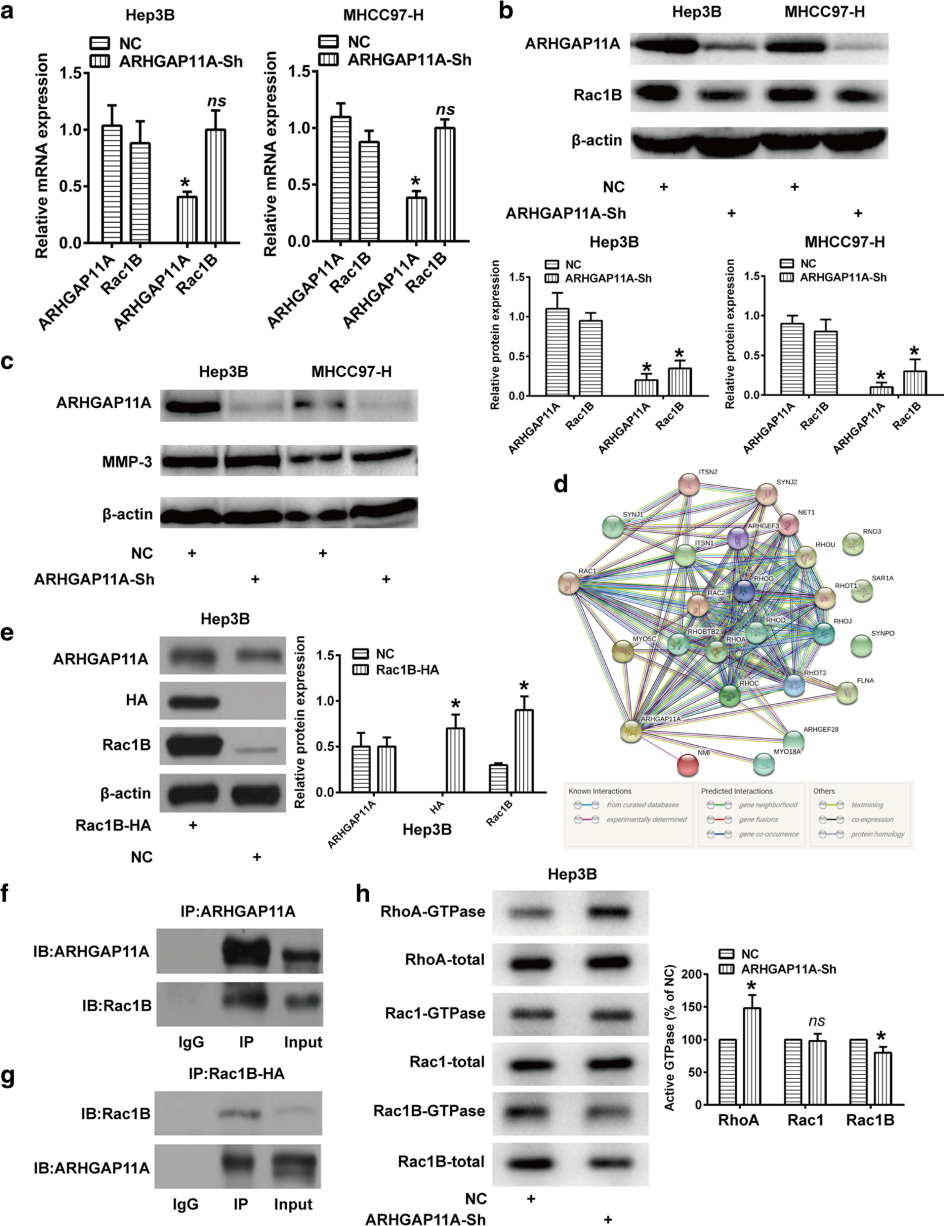
ARHGAP11A directly interacts with Rac1B to exert its roles in HCC. Columns, mean (*n* = 3, in triplicate); bars, SD. *, *P* < 0.05 versus NC. *ns*, no significance. **a** Changes in mRNA expression of Rac1B following ARHGAP11A knockdown in HCC cells. **b** Changes in protein level of Rac1B in ARHGAP11A-knockdown HCC cells or NC. **c** MMP3 protein expression following ARHGAP11A knockdown. **d** GeneCards indicated that ARHGAP11A could directly interact with Rac1. **e** Rac1B expression in Hep3B cells transfected with Rac1B-HA or empty vector (NC) was analyzed with western blotting. **f** and **g** Co-IP assay using Hep3B cells transfected with Rac1B-HA determined the interaction between ARHGAP11A and Rac1B. **h** Blot analysis for GTP-bound RhoA, Rac1/Rac1B levels in cells, with or without ARHGAP11A knockdown following Rho GTPase pulldown experiments. Total protein levels were detected from whole cell lysate

Rac1B is a tumor-associated protein with cell-transforming properties that are linked to matrix metalloproteinase (MMP)-induced EMT in cancers [18]. Previous studies indicated that MMP3 upregulates the expression of Rac1B, which was translocated to the cell membrane to promote EMT [19, 20]. To further elucidate the mechanism involved in ARHGAP11A-induced EMT and malignant phenotype changes, we focused on the MMP-3/Rac1B pathway. Obviously, ARHGAP11A knockdown decreased the protein level of Rac1B, nonetheless, no difference could be found in the mRNA expression (Fig. 5a, b). Meanwhile, ARHGAP11A had no impact on MMP-3 expression (Fig. 5c). Those indicate that ARHGAP11A likely influences Rac1B via posttranscriptional modification rather than translational regulation.

GeneCards (bioinformatics tools, http://www.genecards.org) indicated that ARHGAP11A could directly interact with Rac1 (Fig. 5d). HA-tagged Hep3B cell (Fig. 5e) extraction were used to perform co-immunoprecipitation analyses of ARHGAP11A and Rac1B. As shown in Fig. 5f, ARHGAP11A was firstly immunoprecipitated, and Rac1B could be detected by immunoblot. Then, Rac1B-HA was immunoprecipitated, and ARHGAP11A could also be detected by immunoblot (Fig. 5g). Rho GTPase pulldowns was performed to test GTP-bound RhoA, Rac1, and Rac1B levels. The results showed that ARHGAP11A knockdown increased RhoA, but not Rac1 and Rac1B activity in Hep3B cells. Conversely, ARHGAP11A knockdown decreased total and GTP-bound Rac1B expression (Fig. 5h). All these results revealed that ARHGAP11A and Rac1B interact in HCC cells. More importantly, Rac1B expression was obviously decreased in ARHGAP11A-knockdown cells, suggesting that the MET process caused by ARHGAP11A knockdown was likely regulated by Rac1B.

Considering that ARHGAP11A positively interacted with Rac1B protein, but had no impact on Rac1B mRNA expression, we speculated that ARHGAP11A might affect protein modification of Rac1B in HCC, such as ubiquitination, which was a common form of posttranscriptional modification. Mass spectrometry (MS) also confirmed ARHGAP11A-Rac1B interaction, nevertheless, the ubiquitination of Rac1B was not detected (Additional file 6: Figure S4, Additional file 7: Excel S1).

### Rac1B exerts core role in ARHGAP11A-elicited HCC malignant actions

**Fig. 6:**
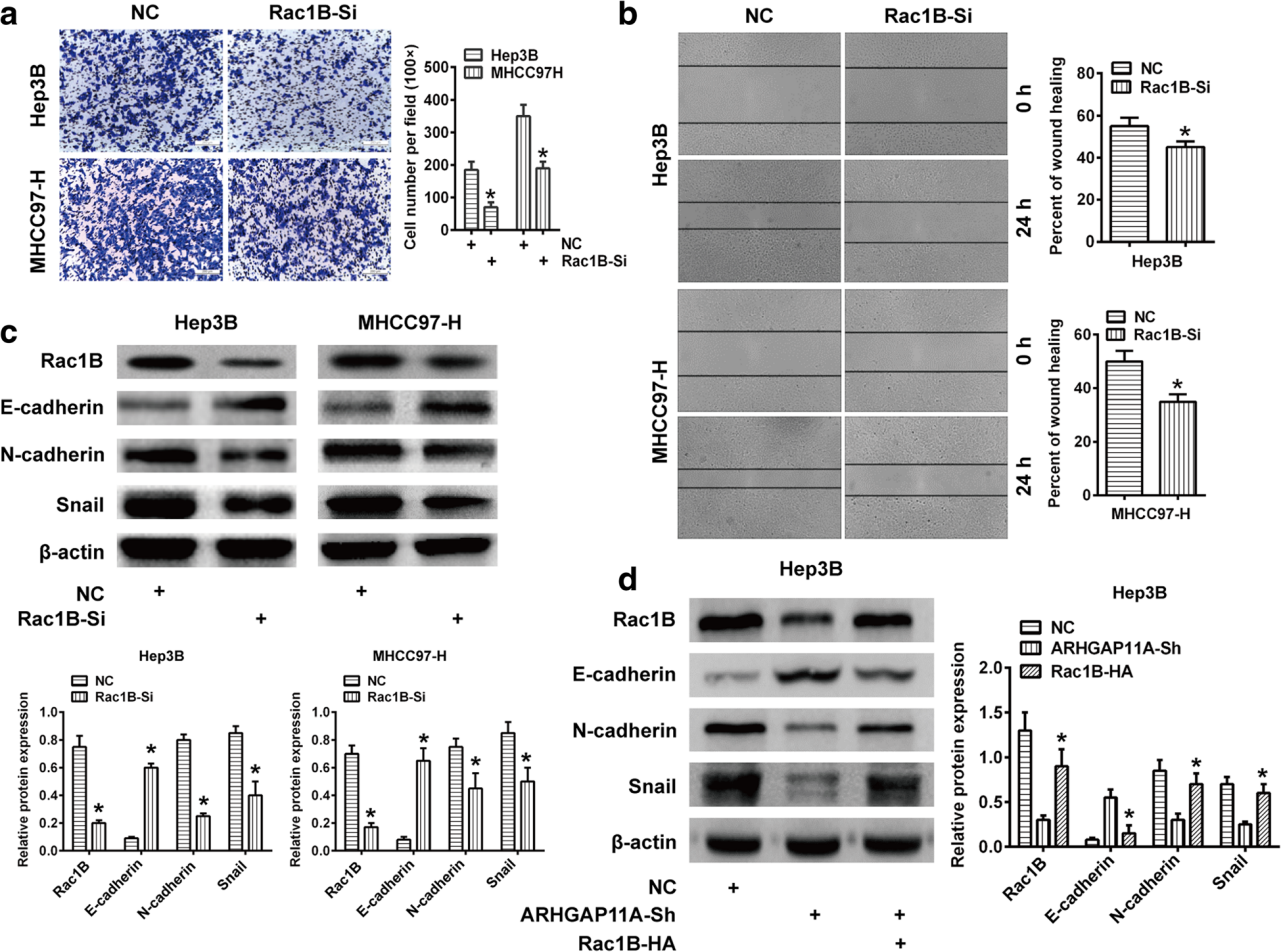
Rac1B is responsible for ARHGAP11A-induced invasion, migration and EMT in HCC. Columns, mean (*n* = 3, in triplicate); bars, SD. *, *P* < 0.05 versus Scrambled (NC). **a** Invasion ability of Rac1B-knockdown HCC cells. Representative images of the Transwell assay are shown. **b** Migration ability of Rac1B-knockdown HCC cells. Representative images of the wound healing assay. **c** The expression of the EMT phenotype markers N-cadherin and Snail was determined by western blot. **d** E-cadherin, N-cadherin and Snail expression in Rac1B-overexpressed ARHGAP11A-Sh Hep3B cells

To further verify that ARHGAP11A promoted HCC EMT, invasion and migration by enhancing the expression of Rac1B, we performed Rac1B gene silencing in Hep3B and MHCC97-H cells by using specific human siRNA. Rac1B silencing significantly reduced the capacity of invasion and migration of HCC cells (Fig. 6a, b). Meanwhile, Rac1B knockdown also led to increased E-cadherin expression and decreased N-cadherin and Snail expression (Fig. 6c). Additionally, we overexpressed Rac1B in ARHGAP11A-shRNA-transfected Hep3B cells, which should result in concurrent ARHGAP11A knockdown and Rac1B overexpression in the same cells. We found that the expression of N-cadherin and Snail was recovered compared with the ARHGAP11A-shRNA group (Fig. 6d). Taken together, these results indicated that Rac1B plays a pivotal role in ARHGAP11A-induced EMT, invasion and migration.

## Discussion

RhoGAPs family, which is well known as regulators of cell migration, invasion and metastasis, has been found downregulation in various cancers [21]. Theoretically, RhoGAPs members are typically classified as growth suppressors and to play inhibitory role in cancer cell proliferation and malignant transformation. Recently, evidences have shown that high expression of RhoGAPs members can be found in some cancers that might serve as oncogenes. SH3BP1 was found to be highly expressed in HCC and could inactivate Rac1 to enhance cell motility [22]. Rho GTPase transcriptome analysis also revealed oncogenic roles for RacGAP1 in basal-like breast cancers [11]. Therefore, RhoGAPs play distinct roles in cancer depending on their spatial regulation and cancer type context [23]. Albeit RhoGAP domain is an evolutionary conserved protein of GTPase activating proteins towards Rho/Rac/Cdc42-like small GTPase, scientists discovered Rho-independent pathway by which some RhoGAPs directly bind to p53 to affect cell proliferation and apoptosis [6]. DLC-1 also showed RhoGAP domain-independent activities [24]. Herein, an uncharacterized RhoGAP, named ARHGAP11A, was investigated in human HCC [25]. We demonstrated that ARHGAP11A expressed in HCC tissue, and showed significant correlation with clinical prognosis in HCC patients. ARHGAP11A knockdown decreased cell proliferation, invasion and migration in HCC in vitro, even metastasis in vivo. Thereby, we have reasons to believe that ARHGAP11A can act as an oncogene, not a tumor suppressor, in human HCC. Our finding was in an agreement with a previous speculation that ARHGAP11A might possess oncogenic and pro-metastatic characteristics [3]. However, Xu et al. also found that ARHGAP11A induced cell cycle arrest and apoptosis via binding to the tumor suppressor p53 in oligodendrocytes [6]. This functional diversity might correlate with cancer type context, and unravel the unique role of ARHGAP11A in HCC. EMT development often appeared during tumor invasion and metastasis which resulted poor prognosis in patients [26]. Evidences showed that EMT facilitated tumor vascular invasion and metastasis in HCC, and contributed to early recurrence [27, 28]. Rho GAPs were found to be involved in EMT regulation in cancers. The CCR2 3′UTR acted as a ceRNA for STARD13 and helped to inhibit cell metastasis by repressing EMT in breast cancer [29]. SH3BP1 is a direct target gene of TAZ in prostate cancer cells, mediating TAZ function in enhancing EMT-meditated cell migration [30]. In present study, we verified that ARHGAP11A induced EMT development in HCC cells, as evidenced by notable morphological changes and phenotype markers transition. E-cadherin was found to be downregulated while N-cadherin and Snail were upregulated, which endow cells with increased motility and invasiveness [31, 32].

Over decades, the role of MMPs in cancer metastasis has been studied [33], and the MMP-3/Rac1B pathway was considered a classic. Rac1B is generated by alternative splicing from *RAC1* also encoding the small GTPase Rac1, a member of the RAS superfamily of small GTP-binding proteins [34]. Rac1B was preferentially overexpressed in malignant lung and breast cancer [35, 36]. In lung cancer, MMP-3 elicited the expression of Rac1B, which subsequently stimulated the expression of transcription factor Snail to induce EMT [20]. Studies have uncovered that Rac1B is crucial for cancer cell proliferation and metastasis [18] and exerted oncogenic activities partly through EMT induction [37]. Rac1B overexpression stimulated Tcf-mediated gene transcription, whereas knockdown of Rac1B resulted in decreased expression of the Wnt target genes C-myc and Cyclin D [38]. Rac1B also reduced E-cadherin expression and cellular adhesion in colorectal cancer cells [39]. Even so, we were not sure about the expression state or exact role of Rac1B in ARHGAP11A-mediated HCC. Thus, we hypothesized that ARHGAP11A might regulate Rac1B to promote HCC growth and EMT development. However, unlike classical MMP-3/Rac1B pathway, there was no change of MMP-3 protein while notable Rac1B reduction could be found in ARHGAP11A-knockdown HCC cells. Inexplicably, qRT-PCR assay indicated that ARHGAP11A had no impact on Rac1B mRNA expression. ARHGAP11A was previously proved to be a GAP specific for Rho, but not for Rac or Cdc42, and ARHGAP11A stimulated cancer cell motility by enhancing Rac activity [10]. Our results also indicated that ARHGAP11A is probably a GAP for RhoA, but not for Rac1 or Rac1B. Though Co-IP assay has confirmed the positive interaction between ARHGAP11A and Rac1B, the regulatory mechanisms by which ARHGAP11A increases Rac1B activity need to be further investigated.

Rac1B was proved to possess enhanced intrinsic guanine nucleotide exchange activity, impaired intrinsic GTPase activity, and failed to interact with Rho-GDP dissociation inhibitors (Rho-GDIs) [40], and the retained GAP-responsiveness alone may not be sufficient to offset the enhanced intrinsic exchange and impaired intrinsic GTPase activities [41]. Thereby, Rac1B was found to exist predominantly in the active GTP-bound state [42]. In our experiment, we speculate ARHGAP11A might impact on Rac1B stability on the premise that ARHGAP11A-knockdown did not result in Rac1B mRNA change. In addition, ARHGAP11A-knockdown apparently affected Rac1B but not Rac1 protein levels, so it is not clear whether ARHGAP11A interacted selectively with Rac1B, but not with Rac1. The Rac1B protein contains an in-frame insertion of 19 amino acids between Rac1 residues 75 and 76 immediately preceding the Switch II region, including two potential threonine phosphorylation sites for casein kinase II and protein kinase C [34], which may alter the intrinsic biochemical properties, as well as interaction with regulators and effectors [41]. Thus, we speculate that Rac1B structural modification may create novel binding sites for ARHGAP11A, albeit more studies will be needed. Recently, a study showed that Rac1B knockdown increased basal ERK activation, and sensitized cells towards further upregulation of phospho-ERK levels by TGF-β1 [37]. However, we did not observe the impact of ARHGAP11A-knockdown on ERK or phospho-ERK expression in our experiments. Therefore, we speculate that EMT in our HCC cells might be TGF-β-independent, which could be explained by differing tumor cells and tumor microenvironments. In the end, our study proved that ARHGAP11A seemed to be GAP specific for RhoA, and can facilitate HCC malignant progress through Rho-independent pathway, albeit more investigations are still needed to ultimately unravel the regulatory mechanism of ARHGAP11A-Rac1B interaction.

## Conclusions

Taken together, our results unravel that ARHGAP11A is frequently upregulated in HCC, and associated with clinical prognosis. ARHGAP11A regulates HCC cell in vitro and in vivo proliferation, migration and invasion, and EMT development via ARHGAP11A/Rac1B pathway, albeit underlying mechanism remains to be fully explored. Our results indicate that ARHGAP11A may be a potential target for the treatment of HCC.

## Additional files

Additional file 1: **Table S1.** Primer sequences used for qRT-PCR. (DOCX 14 kb)

Additional file 2: **Table S2.** Antibodies used for western blotting. (DOCX 16 kb)

Additional file 3: **Figure S1.** Efficacy of ARHGAP11A knockdown in Hep3B and MHCC97-H cells. Columns, mean (*n* = 3, in triplicate); bars, SD. *, *P* < 0.05 versus NC. **a** Expression of ARHGAP11A mRNA in Scrambled (NC) and ARHGAP11A-Sh transfected Hep3B and MHCC97-H cells. **b** Expression of ARHGAP11A protein in NC and ARHGAP1A-Sh transfected Hep3B and MHCC97-H cells. (TIF 379 kb)

Additional file 4: **Figure S2.** Western blot quantification of ARHGAP11A, C-myc, Cyclin D, p-ERK1/2, and ERK1/2 in Hep3B and MHCC97-H cells with or without ARHGAP11A-Sh. (TIF 727 kb)

Additional file 5: **Figure S3.** Expression of P53 protein in Scrambled (NC) and ARHGAP11A-Sh transfected Hep3B and MHCC97-H cells. Columns, mean (*n* = 3, in triplicate); bars, SD. *ns*, no significance. (TIF 365 kb)

Additional file 6: **Figure S4.** Identified proteins’ ubiquitination peptide interacted with ARHGAP11A in HCC (*n* = 3). **a** LEADLEGK from Q5TB80 (CE162). **b** SIGRLSK from Q15032 (R3HD1). **c** SLSKKR from Q7Z4H7 (HAUS6). The b and y ions were indicated with *green and orange colors*, respectively. (TIF 1010 kb)

Additional file 7: **Excel S1.** The proteins interacted with ARHGAP11A in HCC. (XLSX 31 kb)

## Abbreviations

ARHGAP11A: Rho GTPase-activating protein 11A
CCK-8: Cell Counting Kit-8
Co-IP: Co-immunoprecipitation
CoIP-MS: Co-immunoprecipitation coupled with mass spectrometry
EMT: Epithelial-to-mesenchymal transition
HCC: Hepatocellular carcinoma
HE: Hematoxylin and eosin
IPA: Ingenuity Pathway Analysis
MMP: Matrix metalloproteinase
RhoGAPs: Rho GTPase-activating proteins
TCGA: The Cancer Genome Atlas
