# Supplementary material for: Blockade of ARHGAP11A reverses malignant progress via inactivating Rac1B in hepatocellular carcinoma
Source: Cell Commun Signal. 2018 Dec 13;16:99. doi: 10.1186/s12964-018-0312-4 (PMC6293628; doi:10.1186/s12964-018-0312-4)
Supplement: Supplementary file 1 — Table S1. Primer sequences used for qRT-PCR. (DOCX 14 kb) [file 12964_2018_312_MOESM1_ESM.docx]

**Table S1** Primer sequences used for qRT-PCR.

|  | Forward (5’-3’) | Reverse (5’-3’) |
| --- | --- | --- |
| Arhgap11a | GCAGGTGTGCCAAGGCGAAGT | TGCAAGTCGCCAACCAACACTTTCA |
| Rac1B | ATGTCCGTGCAAAGTGGTATC | CTCGGATCGCTTCGTCAAACA |
| Ccnd1 | GCTGCGAAGTGGAAACCATC | CCTCCTTCTGCACACATTTGAA |
| C-myc | GGCTCCTGGCAAAAGGTCA | CTGCGTAGTTGTGCTGATGT |
| Yap1 | TAGCCCTGCGTAGCCAGTTA | TCATGCTTAGTCCACTGTCTGT |
| Nupr1 | CTCTCATCATGCCTATGCCTACT | CCTCCACCTCCTGTAACCAAG |
| β-actin | CTCCATCCTGGCCTCGCTGT | GCTGTCACCTTCACCGTTCC |
