# Supplementary material for: Blockade of ARHGAP11A reverses malignant progress via inactivating Rac1B in hepatocellular carcinoma
Source: Cell Commun Signal. 2018 Dec 13;16:99. doi: 10.1186/s12964-018-0312-4 (PMC6293628; doi:10.1186/s12964-018-0312-4)
Supplement: Supplementary file 2 — Table S2. Antibodies used for western blotting. (DOCX 16 kb) [file 12964_2018_312_MOESM2_ESM.docx]

**Table S2.** Antibodies used for western blotting.

| Primary antibody | Company | Catalog ID | Application(s) and working dilution(s) |
| --- | --- | --- | --- |
| ARHGAP11A | Abcam | #ab113261 | WB 1:1000 IP 5 µg/mg |
| ARHGAP11A | Sigma-Aldrich | HPA040830 | IHC 1:200 |
| E-cadherin | Cell Signaling | #14472 | WB 1:1000 |
| N-cadherin | Cell Signaling | #13116 | WB 1:1000 |
| Snail | Cell Signaling | #3895 | WB 1:1000 |
| Slug | Cell Signaling | #9585 | WB 1:1000 |
| CCND1 | Proteintech | 60186-1-Ig | WB 1:1000 |
| C-myc | Proteintech | 10828-1-AP | WB 1:1000 |
| ERK | Cell Signaling | #4696 | WB 1:1000 |
| p-ERK | Cell Signaling | #9101 | WB 1:1000 |
| MMP3 | Proteintech | 17873-1-AP | WB 1:1000 |
| Rac1B | Millipore | 09-271 | WB 1:1000 |
| HA | Proteintech | 51064-2-AP | WB 1:1000 IP 2µg/mg |
| IgG | Cell Signaling | #9309 | WB 1:1000 |
| β-actin | Proteintech | 60008-1-Ig | WB 1:3000 |
| RhoA | Cell Signaling | #2117 | WB 1:1000 |
| Rac1 | Cell Signaling | #2465 | WB 1:1000 |
