# Supplementary figures and images for: Blockade of ARHGAP11A reverses malignant progress via inactivating Rac1B in hepatocellular carcinoma
Source: Cell Commun Signal. 2018 Dec 13;16:99. doi: 10.1186/s12964-018-0312-4 (PMC6293628; doi:10.1186/s12964-018-0312-4)

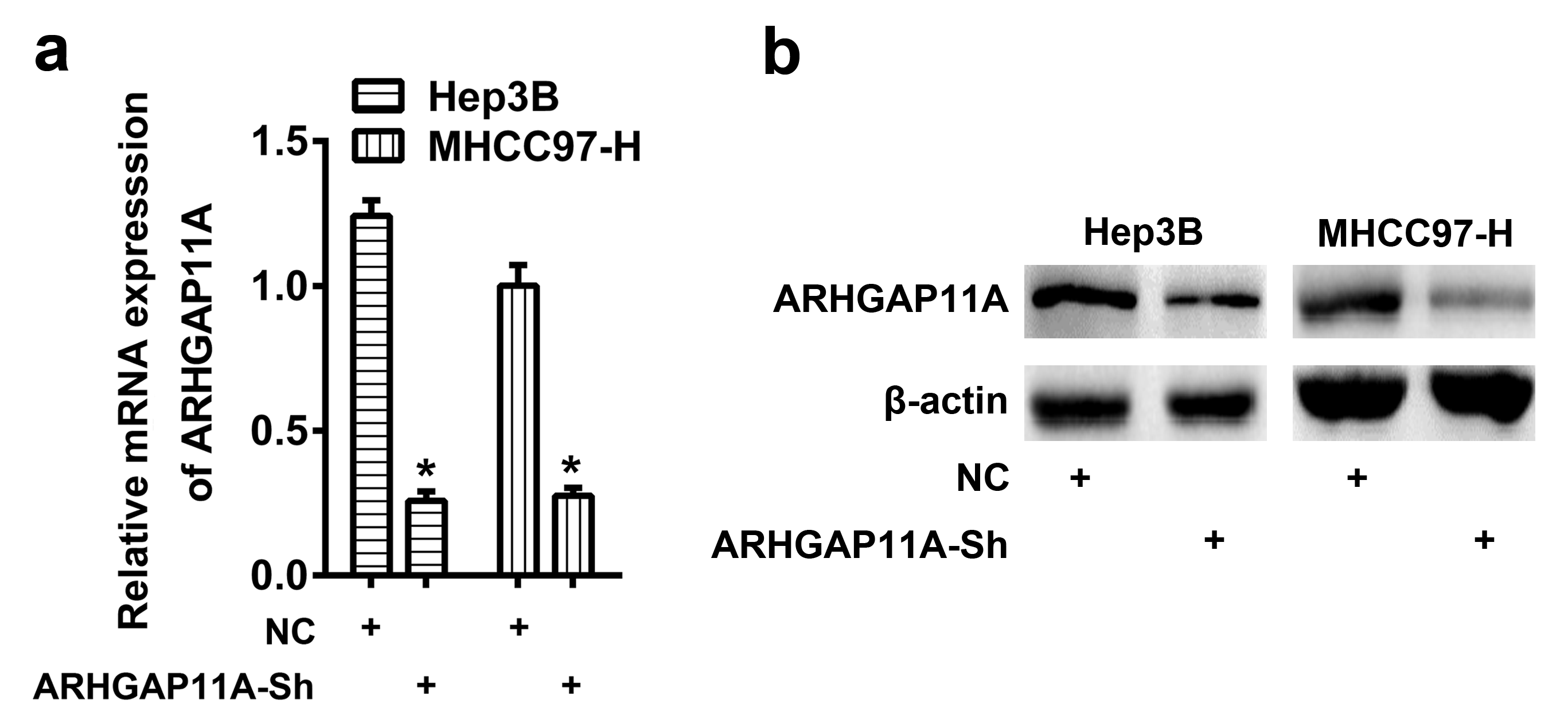

Supplement: Supplementary file 3 — Figure S1. Efficacy of ARHGAP11A knockdown in Hep3B and MHCC97-H cells. Columns, mean (n = 3, in triplicate); bars, SD. *, P < 0.05 versus NC. a Expression of ARHGAP11A mRNA in Scrambled (NC) and ARHGAP11A-Sh transfected Hep3B and MHCC97-H cells. b Expression of ARHGAP11A protein in NC and ARHGAP1A-Sh transfected Hep3B and MHCC97-H cells. (TIF 379 kb) [file 12964_2018_312_MOESM3_ESM.tif]

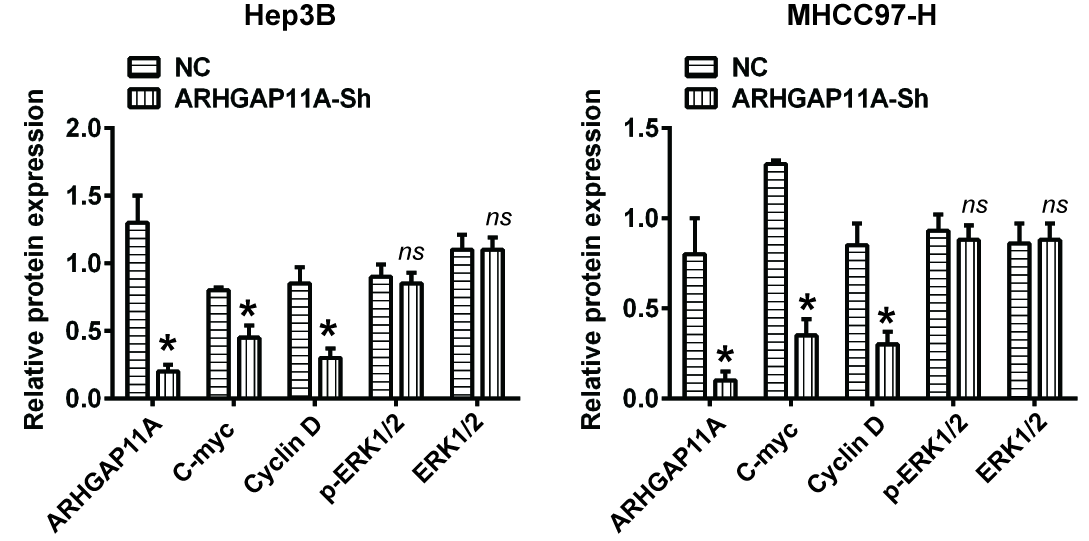

Supplement: Supplementary file 4 — Figure S2. Western blot quantification of ARHGAP11A, C-myc, Cyclin D, p-ERK1/2, and ERK1/2 in Hep3B and MHCC97-H cells with or without ARHGAP11A-Sh. (TIF 727 kb) [file 12964_2018_312_MOESM4_ESM.tif]

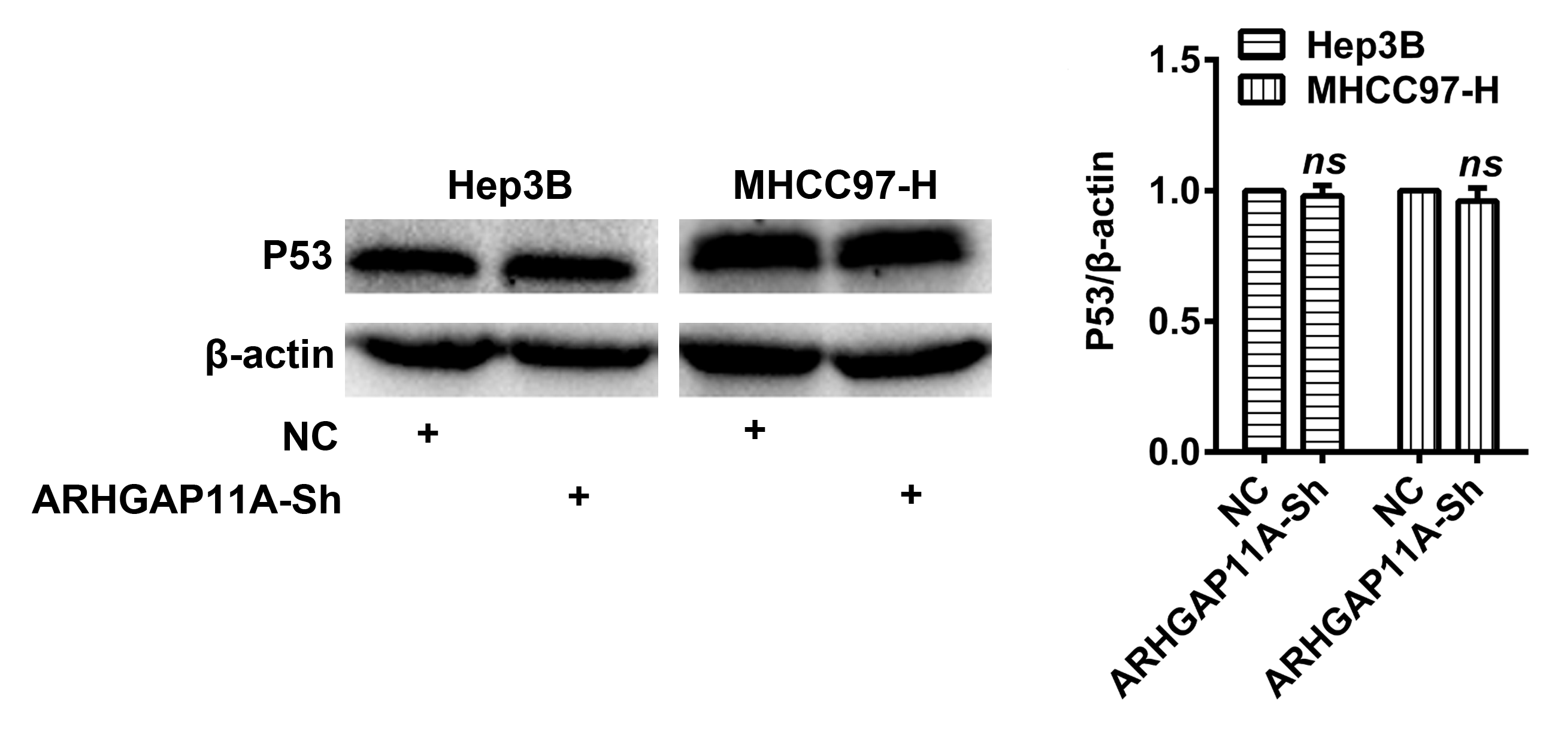

Supplement: Supplementary file 5 — Figure S3. Expression of P53 protein in Scrambled (NC) and ARHGAP11A-Sh transfected Hep3B and MHCC97-H cells. Columns, mean (n = 3, in triplicate); bars, SD. ns, no significance. (TIF 365 kb) [file 12964_2018_312_MOESM5_ESM.tif]

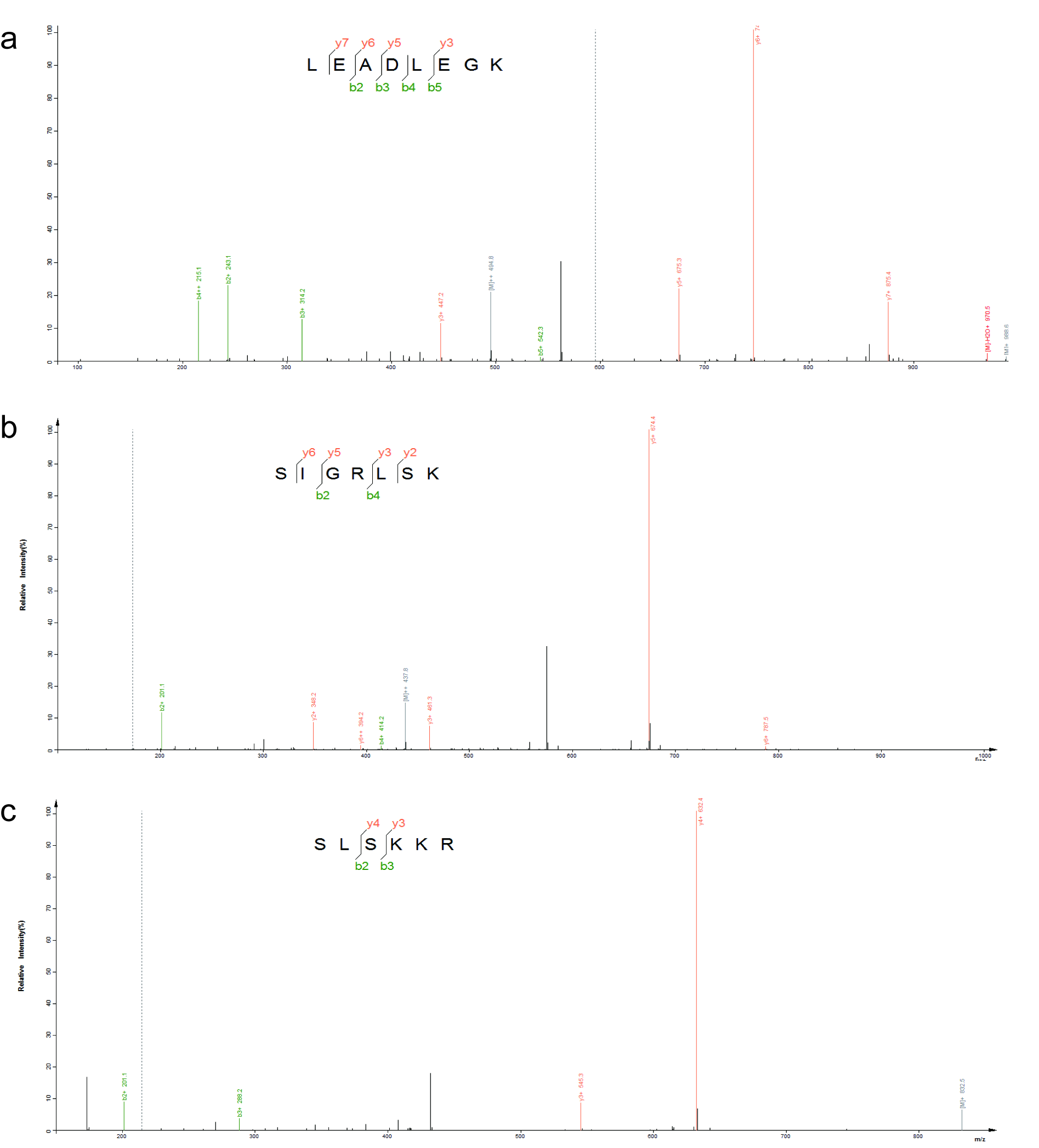

Supplement: Supplementary file 6 — Figure S4. Identified proteins’ ubiquitination peptide interacted with ARHGAP11A in HCC (n = 3). a LEADLEGK from Q5TB80 (CE162). b SIGRLSK from Q15032 (R3HD1). c SLSKKR from Q7Z4H7 (HAUS6). The b and y ions were indicated with green and orange colors, respectively. (TIF 1010 kb) [file 12964_2018_312_MOESM6_ESM.tif]
